# Survey of Resource Scheduling Technologies for Ground-Based Space Target Surveillance Radar Networks Focused on Cataloging Tasks

**DOI:** 10.3390/s26051606

**Published:** 2026-03-04

**Authors:** Yali Liu, Wei Xiong, Xiaolan Yu

**Affiliations:** Graduate School, Space Engineering University, Beijing 101416, China; yaliliu@hgd.edu.cn (Y.L.);

**Keywords:** radar network, cataloging, space target surveillance, multi-objective optimization, task scheduling, resource scheduling

## Abstract

Cataloging task resource scheduling is a key technology for the efficient utilization of ground-based radar networks and for supporting space situational awareness. This problem is highly challenging due to the large scale of tasks, strict time window constraints, and complex resource-task mapping relationships. It requires algorithms to effectively balance multiple conflicting optimization objectives within a huge and sparse solution space, placing extremely high demands on the convergence, diversity maintenance, and computational efficiency of the algorithms. This paper presents a systematic review of the latest research progress in cataloging resource scheduling methods. First, commonly used optimization objectives and constraint conditions in this field are outlined, and two key subproblems—priority modeling and conflict resolution—are analyzed in depth. Subsequently, following the trajectory of technological evolution, the application paradigms, performance characteristics, and limitations of mainstream algorithms are reviewed. Given the inherent multi-objective optimization nature of the problem, the advantages and challenges of multi-objective optimization algorithms are discussed. Finally, based on a unified problem context, the performance and operational boundaries of existing algorithms are compared and analyzed, and future research directions and core challenges in the field are presented.

## 1. Introduction

**Figure 1:**
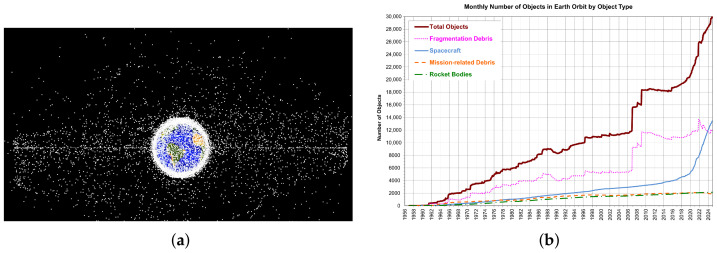
Spatial distribution and growth trend of space objects: (**a**) Spatial distribution of space objects.; (**b**) The growth trend of U.S. cataloged space objects as of 9 January 2025.

With the increasing frequency of space activities worldwide, the number of on-orbit spacecraft and space debris has grown rapidly, leading to a continuous deterioration of the space environment in low Earth orbit (LEO) and a significant rise in collision risks, which seriously threatens the safety of space assets. As shown in Figure 1a, key regions such as LEO and geosynchronous Earth orbit (GEO) have become extremely congested. More critically, as illustrated by the long-term trend in Figure 1b [1], the number of officially cataloged space objects has exhibited an explosive exponential growth. Statistics indicate that there are over 20,000 trackable debris objects larger than 10 cm in LEO, while millimeter-sized debris amounts to hundreds of millions [2]. Given that the average impact speed of space debris reaches 10 km/s, even small debris can gradually degrade spacecraft performance through cumulative effects, and direct impacts from larger debris may lead to complete disintegration and failure of spacecraft. Against this backdrop, building efficient and intelligent ground-based space surveillance systems to achieve accurate space situational awareness and risk early warning has become an imperative for ensuring space security.

However, in the face of the explosive growth in the number of space objects, the contradiction between limited ground-based radar resources and the rapidly growing surveillance demand has become increasingly acute. Developing smarter and more efficient resource scheduling methods has thus emerged as a critical bottleneck in enhancing the protection capabilities of space assets.

Cataloging task resource scheduling serves as the foundation of space target surveillance systems. It is primarily responsible for allocating resources and planning tasks for long-term management missions with known demand information, ultimately generating stable and efficient baseline scheduling plans. Cataloging tasks are characterized by their large scale, complex constraints, sparsely available resources, and significant disparities in task priority. Consequently, resource scheduling for cataloging tasks constitutes a large-scale, strongly constrained NP-hard problem (i.e., computationally intractable for large instances) with a sparse solution space, posing a severe challenge to algorithmic solving capabilities. Furthermore, the quality of a scheduling plan cannot be measured by a single metric; it requires a systematic trade-off among conflicting criteria. How to balance multiple objectives (such as task completion rate, coverage rate for high-value targets, and resource load balancing) is also a critical issue that demands careful consideration.

Currently, researchers have developed various algorithms for cataloging task resource scheduling, including exact solution algorithms [3,4], heuristic algorithms [5,6,7,8,9], and metaheuristic algorithms [10,11,12]. In recent years, with the rapid advancement of Artificial Intelligence (AI) technologies, intelligent algorithms such as neural networks, reinforcement learning (RL), and their derivative deep reinforcement learning (DRL) have demonstrated strong adaptive and decision-making capabilities in the field of resource scheduling [13,14,15,16,17], gradually becoming a research hotspot. At the macro level, the literature [18] provides a systematic review of the development of space situational awareness, but primarily focuses on the overall architecture and technological evolution, without delving into resource scheduling problems in large-scale solution spaces. In the adjacent field of spacecraft TT&C task scheduling, the study [19] comprehensively compares problem classification, modeling, and solution algorithms, offering important references for window-based resource scheduling. However, none of the above studies target cataloging task scenarios; they fail to cover key characteristics such as the massive task scale, sparse feasible solutions, and heterogeneous radars (mechanical/phased-array), nor do they provide empirical comparisons of different algorithms under a unified benchmark. To fill this gap, this paper presents the first systematic review of ground-based radar network resource scheduling for cataloging tasks. The main contributions of this study are threefold, as follows:

- A Systematic Synthesis: This work presents a systematic review of ground-based radar network resource scheduling for cataloging tasks. It comprehensively organizes problem models, constraints, and optimization objectives, while dissecting two core and persistent decision-making issues: priority modeling and conflict resolution. Subsequently, it systematically traces the complete algorithmic development context from single-objective optimization (including exact algorithms, heuristic algorithms, metaheuristic algorithms, and AI algorithms) to multi-objective optimization, clearly illustrating the capability evolution and paradigm shift of solution technologies in this field.
- A Unified Empirical Benchmark: A standardized multi-scenario simulation benchmark is established. For the first time, the performance boundaries of mainstream algorithms are empirically compared under a unified framework, revealing the performance degradation patterns and key bottlenecks of various algorithms as the task scale expands.
- Pathways for Integrated Innovation: Based on empirical results, it is clearly pointed out that the domain knowledge-embedded hierarchical optimization framework is an effective paradigm for balancing scheduling quality and efficiency. Furthermore, future integrated innovation directions such as integrating AI and multi-objective optimization decision support modules are proposed, providing a clear roadmap for breaking through the bottlenecks of large-scale complex scheduling in this field.

**Figure 2:**
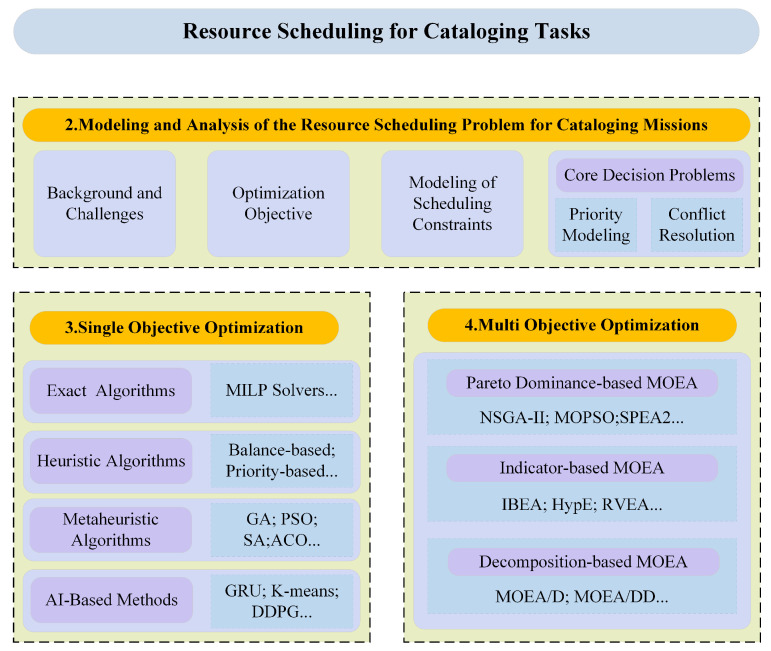
The organizational structure of this review.

The organizational framework of this review is illustrated in Figure 2. Section 2 provides an analysis of the resource scheduling problem for cataloging-oriented space object surveillance. It outlines the commonly used optimization objectives and scheduling constraints in current research, and examines two core decision-making issues: priority modeling and calculation methods, as well as resource conflict and conflict resolution methods. Section 3 and Section 4 summarize the characteristics and operational boundaries of algorithms currently applied to single-objective cataloging resource scheduling and multi-objective cataloging resource scheduling, respectively. Section 5 selects representative heuristic and metaheuristic algorithms to solve the cataloging task resource scheduling problem. It analyzes and compares the strengths and applicability limits of several algorithms, and discusses the limitations of current research along with feasible future directions. Finally, the various resource scheduling algorithms mentioned in this review are summarized.

## 2. Review Methodology

To ensure the comprehensiveness, systematicity, and reproducibility of this review, we followed the Preferred Reporting Items for Systematic Reviews and Meta-Analyses (PRISMA) guidelines [20] to construct the screening process and analytical framework for the literature collection.

### 2.1. Literature Search Strategy

To comprehensively cover research in the field of resource scheduling for ground-based space target surveillance radar networks, we formulated a multi-level retrieval strategy to mitigate potential coverage bias.

Search databases: A comprehensive search was conducted in IEEE Xplore, Web of Science, Scopus, EI Compendex, and China National Knowledge Infrastructure (CNKI).

Search keywords: The search query was constructed around three core concepts:

- Subject terms: “space target” OR “space object” OR “cataloging”;
- Resource terms: “radar network” OR “phased-array radar” OR “ground-based radar”;
- Problem terms: “resource scheduling” OR “task scheduling” OR “mission planning”.

Supplementary search: Backward citation chasing (snowballing) was performed on the references of included studies, and a manual search of top journals and conferences in the field over the past 5 years was conducted to minimize omissions.

### 2.2. Inclusion and Exclusion Criteria

Inclusion criteria:

- The research topic is resource scheduling or TT&C resource scheduling for space targets using ground-based radar;
- The research scenario is oriented toward cataloging tasks (routine, long-term management);
- The document type is journal article, official website publication, dissertation, or conference paper;
- The language is English or Chinese.

Exclusion criteria:

- Studies that only discuss signal processing or target tracking algorithms without addressing high-level scheduling;
- Studies that only target dynamic scheduling for emergency response or new object discovery;
- Studies that do not involve resource scheduling with visibility time windows.

### 2.3. Literature Screening Process

**Figure 3:**
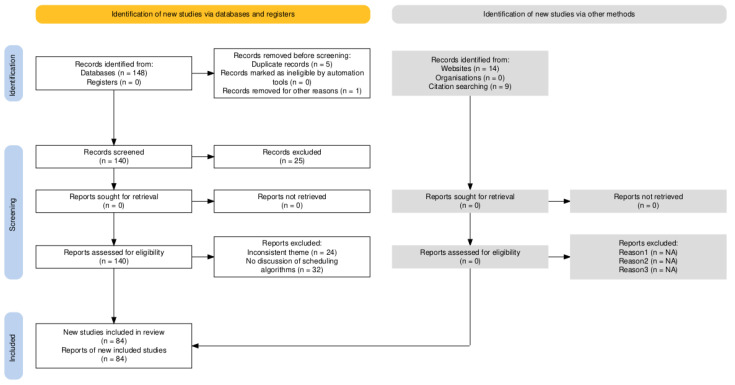
The literature screening flowchart.

The literature screening flowchart was constructed using the tool provided by Haddaway [21], as shown in Figure 3. The final 84 studies identified through the PRISMA process constitute the core literature for this review, supplemented by additional methodological references such as the PRISMA guideline itself [20].

- A total of 148 records were obtained from database searches, and an additional 23 records were identified through other sources;
- After deduplication and removal of 6 records, 165 records remained;
- After initial screening of titles and abstracts, 25 records were excluded, leaving 140 records for full-text review;
- After full-text review, 56 records were excluded, and 84 studies were finally included in the core analysis.

## 3. Modeling and Analysis of the Resource Scheduling Problem for Cataloging Missions

### 3.1. Background and Challenges

The complexity of resource scheduling for cataloging-oriented space target surveillance stems from the inherent conflict between large-scale task demands and limited system resources. From a technical perspective, the tasks involved in the system (including detection, tracking, identification, orbit determination, and cataloging) can be abstracted into two basic modes: “search” and “tracking” [22]. From the scheduling standpoint, the core task of the system is to achieve efficient resource allocation in a multi-target tracking environment. Given the limited radar resources and overlapping visibility time windows of different targets, this inevitably leads to fierce competition for time resources and severe resource conflicts. In summary, resource scheduling for cataloging tasks currently faces the following challenges:

1. Severe Resource Conflicts and Constraint Complexity. The system confronts the fundamental dilemma of “numerous targets versus limited resources”. Moreover, the strict visibility time windows, which are dictated by factors such as orbital mechanics, geospatial geometric relationships, and the inherent physical properties of radar equipment, further compound the difficulty of resource allocation. These windows are short-lived, sparse, and mutually overlapping; they fragment available resources into numerous discrete segments, making the construction of a globally feasible scheduling plan inherently challenging.
2. NP-hard Computational Complexity Induced by Problem Scale. Matching fragmented windows with a massive number of tasks leads to an exponential growth in the solution space, which poses severe challenges to the convergence and global optimization capabilities of algorithms. This fundamentally renders the search for a global optimal solution computationally infeasible. Furthermore, the large task scale results in a sharp increase in computational resource consumption, imposing higher requirements on the convergence speed of algorithms. How to balance solution quality and computational efficiency constitutes a key issue that needs to be prioritized in cataloging task scheduling.
3. Multi-objective Trade-offs and Decision Support Dilemmas. The evaluation of a cataloging task scheduling scheme cannot rely on a single performance metric. Instead, it requires systematic trade-offs among conflicting criteria, such as task completion rate, high-value target assurance rate, and resource load balancing. A key challenge, therefore, lies in how to better balance these competing objectives and provide decision makers with a diverse set of scheduling options. Furthermore, the Pareto front output by optimization algorithms contains a large number of nondominated solutions. How to assist decision makers in making rapid and scientific final decisions also demands significant attention.

### 3.2. Mathematical Formulation of the Problem

#### 3.2.1. Definition of Symbols

We define the following sets, parameters, and decision variables.

T: the set of cataloging tasks, containing all *V* tasks.
Each task i∈T (denoted as $task_{i}$) has four attributes:
  $est_{i}$: earliest start time,
  $let_{i}$: latest end time,
  $p_{i}$: priority (benefit of completion),
  $tracktime_{i}$: minimum required tracking duration.
The task must be executed within $\left[est_{i},let_{i}\right]$. Its contribution is determined by $p_{i}$.
TW: the set of visibility windows between all space targets and each radar in the ground-based radar network.
Each visibility window tw∈TW (denoted as $tw_{ij}$) contains four attributes:
  *j*: radar index,
  *i*: corresponding space target index,
  $st_{ij}$: start time,
  $et_{ij}$: end time.
Every $tw_{ij}$ uniquely associates a radar and a space target; the effective visibility between $target_{i}$ and $radar_{j}$ determines $st_{ij}$ and $et_{ij}$.
R: the set of ground-based radars.
Each radar j∈R (denoted as $radar_{j}$) has two attributes:
  $abi_{j}$: multi-target surveillance capability: the maximum number of space targets that radar *j* can track simultaneously at the same moment.
  $act_{j}$: state transition time: the time required for an independent beam of radar *j* to adjust its pointing between two consecutive tasks.
Decision Variables:
  $x_{ij}\in \left\{0,1\right\}$: $x_{ij}=1$ if task $task_{i}$ is executed on radar $radar_{j}$, and $x_{ij}=0$ otherwise.
  $s_{ij}\in R^{+}$: start time of task $task_{i}$ on radar $radar_{j}$ (meaningful only when $x_{ij}=1$).
By determining the assignment $\left\{x_{ij}\right\}$ and the start times $\left\{s_{ij}\right\}$ for each task on each radar, the final resource scheduling plan for space target cataloging tasks is obtained.

#### 3.2.2. Basic Assumptions

To transform the complex space object cataloging problem into a scientific problem amenable to mathematical modeling, we make the following assumptions:

1. Normal equipment operation. All equipment operates normally; task failures caused by equipment malfunctions or other factors are not considered.
2. Non-preemptive execution. Once a surveillance task starts, the resource it occupies will not be preempted by other tasks.
3. Time-only resource scheduling. Only the time resource of radars is scheduled; other factors such as power and frequency are not considered.
4. LEO-only catalog maintenance. Only catalog maintenance tasks for existing LEO targets are considered; new tasks and MEO/GEO targets are not considered.
5. Simplified visibility analysis. When analyzing visibility between radars and space targets, only geometric visibility and ground clutter effects are considered.

#### 3.2.3. Core Constraints

(1) $$\sum_{j=1}^{J}x_{i,j}\leq 1,\;\forall i\in \left[1,V\right]$$

(2) $$st_{i,j}\leq s_{i,j}\leq s_{i,j}+{\mathrm{tracktime}}_{i}\leq et_{i,j}$$

(3) $$est_{i}\leq s_{i,j}\leq s_{i,j}+{\mathrm{tracktime}}_{i}\leq let_{i}$$

(4) $$s_{i,j}+{\mathrm{tracktime}}_{i}+{\mathrm{act}}_{j}\leq s_{i+1,j}$$

(5) $$\sum_{i\in T}1_{\left[s_{i,j},s_{i,j}+{\mathrm{tracktime}}_{i}\right]}\left(t\right)\leq abi_{j},\;\forall j\in R,\forall t\in \left[0,T\right]$$

where $x_{i,j}$ indicates whether task *i* is executed on resource *j*, *V* denotes the total number of cataloging tasks to be scheduled, $1_{\left[s_{i,j},s_{i,j}+{\mathrm{tracktime}}_{i}\right]}\left(t\right)$ denotes an indicator function that takes a value of 1 if time t∈[0,T] (where *T* represents the end time of the resource scheduling horizon with the start time of scheduling defined as time 0) lies within the execution interval of task *i* on radar *j*, and 0 otherwise. Equation (1) states that each task can be assigned to at most one radar; Equation (2) requires that the task execution time must start after the start time and finish before the end time of the selected visibility window; Equation (3) ensures that the task execution time must start after its earliest allowed start time and finish before its latest allowed end time; Equation (4) imposes that a sufficient state transition time must be reserved between two consecutive tasks executed on the same independent beam; Equation (5) enforces that, at any moment, the number of tasks simultaneously tracked by radar *j* does not exceed its multi-target surveillance capability ${\mathrm{abi}}_{j}$.

#### 3.2.4. Optimization Objectives

Cataloging task resource scheduling is inherently a multi-objective optimization problem. Different studies select different combinations of objectives according to their application scenarios. In general, the optimization objectives can be categorized into two dimensions: task satisfaction and resource utilization.

Task satisfaction is commonly measured by indicators such as task completion rate, total priority score, number of completed tasks, or critical task completion rate. Resource utilization is often evaluated through load balancing degree, resource occupancy rate, or ground station priority.

A generic multi-objective formulation can thus be expressed as

$$\max\;\left(f_{\mathrm{satisfaction}},\;f_{\mathrm{utilization}}\right)$$

where $f_{\mathrm{satisfaction}}$ and $f_{\mathrm{utilization}}$ represent specific metrics from the above categories.

This vector form is only an exemplary abstraction. The actual choice and formulation of objectives vary considerably across the literature; a detailed summary of the commonly adopted objectives is provided in Section 3.4 and is therefore not repeated here. In practice, decision makers may either select a single objective or optimize multiple objectives simultaneously according to specific mission requirements.

#### 3.2.5. Computational Complexity

The above optimization problem is NP-hard in general. Its difficulty stems from the coupling of the following three structural factors:

1. Combinatorial Explosion of Assignment and Scheduling
  The assignment of tasks to radars ($x_{ij}$) and the determination of start times ($s_{ij}$) must be decided jointly. The size of the solution space grows super-exponentially with the number of tasks *V*, radars *J*, and the average number of visible windows *K* ($\Omega ({\left(J\cdot K\right)}^{V}\cdot \prod_{j}n_{j}!)$), where $n_{j}$ is the number of tasks assigned to radar *j*.
2. Resource Fragmentation Due to Time Windows
  Visible windows fragment each radar’s available time into discontinuous and overlapping segments. Even without considering priorities, scheduling a set of tasks within non-conflicting windows while meeting deadlines is itself an NP-hard subproblem.
3. Heterogeneity and Coupling of Radar Networks
  The complexity increases further when the radar network comprises both mechanical radars ($abi_{j}=1$, single-target tracking with strict non-overlapping and state transition time requirements) and phased-array radars ($abi_{j}\geq 2$, multi-target tracking with instantaneous capacity constraints):
  - Although the feasibility subproblem on a mechanical radar (with a fixed set of tasks) can be solved in polynomial time, this subproblem is tightly coupled with the global assignment and sequencing decisions; its tractability does not reduce the overall NP-hardness.
  - For a phased-array radar, even if the assignment decision is temporarily set aside and we consider only a single radar with a fixed set of tasks, the problem of determining whether there exists a set of start times that satisfies both the time window constraints and the instantaneous capacity ${\mathrm{abi}}_{j}$ is itself NP-hard. This means that each additional phased-array radar introduces an inherently intractable substructure into the system.
  - When multiple radars of different types coexist, tasks can be freely assigned across different radar types. The feasibility subproblems of individual radars are coupled through the shared task set. This coupling exacerbates resource competition and constraint interaction, making the construction of feasible solutions significantly more challenging compared with scenarios with a single radar type.

This complexity analysis indicates that exact algorithms are only viable for small instances. For practical radar network scenarios, approximate methods such as heuristics, metaheuristics, and deep reinforcement learning are essential. This analysis provides the theoretical foundation for the subsequent discussion on algorithm classification and evolution.

### 3.3. Engineering Perspective and Classification of Scheduling Constraints

Section 3.2.3 formulates the core constraints of cataloging task scheduling in a mathematical form. However, practical engineering scenarios often involve additional constraints, and the modeling granularity varies across different studies. Table 1 summarizes the various types of constraints that appear in the literature of this field, along with their engineering implications.

**Figure 4:**
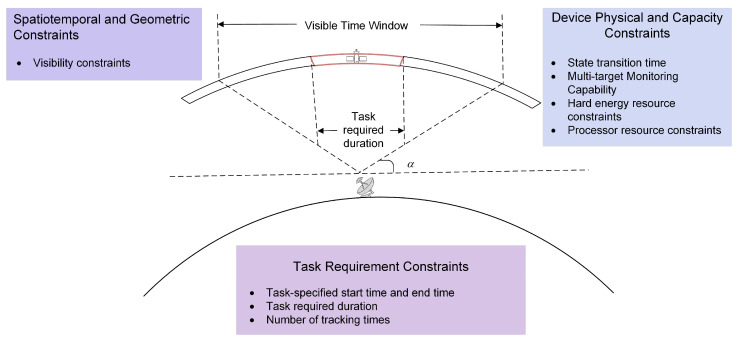
Schematic diagram of resource scheduling constraints.

Among these constraints, the visibility of space targets to surveillance resources is a prerequisite for space target surveillance. The geometric visibility between ground-based radars and space targets is limited by the radars’ inherent capabilities (e.g., elevation angle, azimuth angle, and maximum detection range). Geometric visibility serves as a basic condition for space target surveillance; however, not all geometrically visible arcs enable effective surveillance (e.g., affected by ground clutter, the surveillance performance of radars on space targets becomes unacceptable when the elevation angle between the radar and the target is less than a certain threshold). Therefore, to ensure surveillance effectiveness, the minimum elevation angle for visibility time windows is usually set to α, and ground-based radars only regard time windows where the elevation angle with space targets is greater than α as valid visibility time windows. Figure 4 illustrates the constraints that need to be considered for the successful execution of space target surveillance tasks.

### 3.4. Engineering Perspective and Classification of Optimization Objective

As described in Section 3.2.4, the cataloging task resource scheduling problem is essentially a multi-objective optimization problem, and its optimization objectives can be summarized into two dimensions: task satisfaction and resource utilization. This section further provides a systematic review of the specific implementations of these two types of objectives in the existing literature. Currently, relevant studies on cataloging task scheduling mainly focus on improving task satisfaction by enhancing the task satisfaction rate or quality. This is achieved through the optimization of performance aspects such as total priority score [23], request failure rate [24], weighted sum of squared optimal time offsets of tasks [25], and target assignment efficiency [26]. In contrast, relatively few studies have focused on resource utilization, which mainly involves reducing the switching frequency of radar networks [27], comprehensively considering target priority, equipment load balancing, and task arrangement rationality [28], and introducing equipment load balancing allocation rules [5]. However, in practical application scenarios, the rationality of resource utilization often determines the service life and failure probability of radar networks, which requires prioritized attention. To achieve a comprehensive understanding of the optimization objectives of scheduling models, it is necessary to not only focus on mainstream task satisfaction indicators but also fully consider the rationality of resource utilization. To this end, Table 2 systematically summarizes the mainstream optimization objectives and their implications in this field, aiming to provide a reference for the selection and design of optimization objectives in subsequent studies.

### 3.5. Core Decision Problems

#### 3.5.1. Priority Modeling

In the space domain, there exists an inherent contradiction between the vast number of space objects and the limited availability of ground-based surveillance resources. This leads to intense competition for resources among tasks, posing a significant challenge for decision makers when formulating scheduling plans. However, not all space objects possess equal strategic value or require the same level of monitoring intensity. To maximize the utilization of limited resources, a surveillance system must be capable of accurately identifying and prioritizing the protection of key targets that pose a direct threat to space security or hold high value. Examples include critical operational assets in orbit, objects with a high risk of collision, and those exhibiting potential for anomalous state changes.

Against this backdrop, the concept of priority has been introduced as a core metric for quantifying the relative importance of targets. It serves not only as the basis for decision making in the competitive allocation of resources but also as a crucial bridge for translating macro-level strategic requirements into specific scheduling directives. Establishing a scientific and rational priority modeling framework has thus become an essential prerequisite and a foundational cornerstone for enhancing the effectiveness of ground-based space object surveillance systems and ensuring the security of national space assets.

The quantification of space object priority is a typical multi-attribute decision-making problem. As shown in Table 3, numerous influencing factors are involved, spanning three major dimensions: target attributes, task attributes, and system status. In practical modeling, key indicators must be selected from these dimensions based on specific task scenarios and decision-making preferences for comprehensive integration.

Currently, mainstream research integrates the influencing factors of priority into a unified scalar value primarily through the following three categories of methods:

1. Weighted Summation. This method is simple, intuitive, and computationally efficient, making it the most widely applied priority calculation approach. However, its performance heavily relies on the subjective assignment of weights and struggles to accurately capture the complex nonlinear couplings between different factors.
2. Multi-Attribute Decision Making (MADM). This category includes methods such as the Technique for Order Preference by Similarity to an Ideal Solution (TOPSIS) and the Analytic Hierarchy Process (AHP). By constructing judgment matrices or calculating relative closeness to ideal solutions, these methods can handle qualitative indicators and weight allocation more scientifically. They are particularly suited for decision scenarios involving numerous factors where minimizing subjective arbitrariness is crucial.
3. Data-Driven Methods. This paradigm abandons manually designed formulas and instead automatically learns priority strategies from historical data or interactive simulations. It can implicitly capture the complex mapping relationships between influencing factors and the final priority, holding theoretical potential to address the most complex scenarios and representing a frontier research direction in the field. However, its application is limited by the explainability challenges arising from the “black-box” nature of its decisions, as well as its dependence on large volumes of high-quality training data.

The aforementioned calculation methods, when dealing with different types of priority influencing factors, naturally give rise to two distinct modeling paradigms: static priority and dynamic priority. Their fundamental distinction lies in whether the priority assignment dynamically changes as a function of system state and time.

1. Static Priority Model. The static priority model mainly depends on a target’s intrinsic attributes (e.g., strategic affiliation, target function). It is uniquely determined when a task is initiated and remains fixed for the entire duration of the scheduling cycle. Boasting high computational efficiency, this model provides a stable, predictable decision-making basis for scheduling systems, and has therefore been widely adopted in early-stage studies and systems with stringent stability requirements. For instance, static attributes (e.g., strategic affiliation, RCS) are integrated using the AHP [29], or priorities are manually assigned by analyzing target functions and task types [30]. However, as the model cannot adapt to dynamic changes in the task execution environment, it is prone to priority inversion. This issue arises when a high-value task fails to be executed: its available time window narrows to the point that the task cannot be completed, thereby undermining the system’s overall performance.
2. Dynamic Priority Model. To overcome the inherent limitations of the static model, the dynamic priority model has emerged. By incorporating time-varying factors, such as the remaining time to deadline and the time since last observation, it enables the priority to adaptively adjust based on the actual situation. This significantly enhances the rationality and scientific rigor of the priority model. For instance, reference [7] constructs a time-varying dynamic priority by weighting and aggregating factors like task threat level, dwell time, and deadline. This approach effectively guides resources toward critical and urgent targets, markedly improving the scientific basis and responsiveness of scheduling solutions. However, this gain comes at the cost of frequent re-evaluations and increased computational overhead, which can subsequently impact the scheduling efficiency for cataloging tasks.

In summary, space object priority modeling reflects a shift from static description to dynamic perception. This evolution enables models to integrate attributes across more dimensions and respond to environmental changes, thereby significantly enhancing the scientific rigor of decision making. However, its development remains constrained by the inherent contradiction between decision depth and computational real-time requirements. Therefore, the focus of future research should not be the unlimited complication of models. Instead, efforts should be directed toward exploring lightweight dynamic models or heterogeneous hybrid frameworks that combine static and dynamic approaches. The goal is to achieve a precise balance among model completeness, environmental adaptability, and decision-making timeliness.

#### 3.5.2. Conflict Resolution Strategies

**Figure 5:**
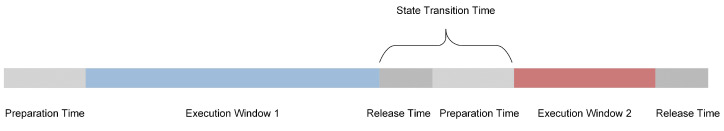
State transition time diagram.

Resource scheduling for cataloging tasks is, in essence, a competitive allocation of limited observation resources under multi-dimensional constraints such as time and tracking capability. Resource conflict is an inevitable core issue in this process, manifesting primarily in two aspects: first, competition among tasks for resources, i.e., multiple space targets being simultaneously visible to a single ground-based radar; second, inherent physical limitations of the radar itself, i.e., the radar has a finite capacity for simultaneously tracking space targets, and its beam must satisfy a certain state transition time constraint when switching between two consecutive tasks (as shown in Figure 5). These two types of conflict together constitute the core conflicts within the scheduling model, and the performance of the strategies employed to resolve them is directly related to the feasibility and effectiveness of the final scheduling solution.

Through reviewing and analyzing the relevant literature [31,32,33,34,35,36,37,38,39,40,41,42], the conflict resolution methods are categorized into the following classes, as summarized in Table 4.

Among the three aforementioned conflict resolution strategies, the Selection-Elimination Method embodies the principle of efficiency first, the Conflicting Window Pruning Method represents the concept of flexible negotiation, while the Conflict Backtracking Class pursues the achievement of a globally optimal solution whenever possible. In practical applications, different methods can be combined. For instance, the simplest and most efficient Selection-Elimination Method can be used in scheduling scenarios with high real-time requirements, whereas the Conflict Backtracking Class can be chosen for scenarios prioritizing global optimal performance. Future work could focus on the development of an adaptive conflict resolution framework, enabling the system to intelligently select or switch between appropriate conflict resolution strategies based on factors such as conflict scale, task real-time requirements, and available computational resources.

## 4. Single Objective Optimization-Based Conventional Cataloging Resource Scheduling

### 4.1. Exact Algorithm

Exact algorithms are a class of algorithms with fixed solution steps that can find the optimal solution or prove the problem has no solution within a certain time and space complexity. In the early stages, when the number of tasks and resources was limited, exact algorithms could obtain optimal resource scheduling schemes within a reasonable amount of time. Therefore, many early researchers attempted to use various exact algorithms to solve resource scheduling problems, contributing effective ideas. Examples include using a network flow-based two-stage decision model to solve a single-objective, multi-resource measurement and control scheduling problem [3], developing Lagrangian-based repair and relaxation heuristic models for Mixed-Integer Programming (MIP) to solve complex constrained scheduling problems [4], and using the Alternating Direction Method of Multipliers (ADMM) to address the issues of time resource scarcity and resource competition in multi-task scheduling for phased-array radars [43]. However, as an NP-hard problem, with the rapid expansion of the task scale, the solution space, solution difficulty, and computational complexity all increase exponentially. Exact algorithms have become inadequate for tackling such problems.

### 4.2. Heuristic Algorithm

The cataloging-oriented radar network resource scheduling problem is a typical NP-hard problem, for which obtaining the exact optimal solution in polynomial time is challenging. To address the growing scale of routine cataloging resource scheduling, researchers have increasingly adopted heuristic algorithms, valued for their high computational efficiency and ease of engineering implementation [44,45]. This shift has been characterized by a clear evolutionary trend: from simple early-stage rules to combinations of multiple rules, and ultimately to dynamic adaptive strategies. To systematically elaborate on this evolutionary process and facilitate a clear comparison of different heuristic algorithms, the core characteristics, application scenarios, and pros and cons of each stage are summarized in the following Table 5.

In summary, to address the increasingly large-scale and complex evolving task scenarios, heuristic algorithms have undergone a technical evolution from single indicators to multi-indicators and from static indicators to dynamic indicators. Their characteristics of high computational efficiency, intuitive rules, and integration of domain knowledge for guidance enable them to stably generate feasible solutions in cataloging task scheduling. However, their inherent drawbacks, including local optimality, difficulties in multi-indicator balancing, and poor adaptive capability, are particularly prominent in large-scale, complex, and dynamically changing scheduling scenarios. To further improve the global optimality, multi-indicator trade-off capability, and adaptability to complex and variable scenarios of scheduling solutions, researchers have introduced more sophisticated and intelligent metaheuristic algorithms and AI-based algorithms into this field.

### 4.3. Metaheuristic Algorithm

Metaheuristic algorithms search for solutions to problems by simulating biological evolution, group cooperative behaviors, and physical phenomena in nature, exhibiting strong adaptability and self-organization. In recent years, with the continuous growth in the number of space targets, metaheuristic algorithms have gradually emerged as an important solution for resource scheduling problems by virtue of their advantages in swarm search and global optimization [10,11,12]. The difficulties in cataloging task scheduling stem from the explosion of the decision space and the sparsity of feasible solutions; therefore, the improvement direction of algorithms lies in enhancing search capability to further improve solution quality. To address these challenges, researchers have proposed various enhancement strategies for metaheuristic algorithms, which are summarized in Table 6.

In summary, metaheuristic algorithms for cataloging task scheduling have consistently aimed to address the core challenges posed by scale explosion and sparse feasible solutions, continuously improving solution quality and optimization efficiency through various improvement strategies. However, with the continuous escalation of tasks in terms of scale and complexity, the limitations of heuristic and metaheuristic algorithms have gradually become prominent: the former, characterized by local optimality, difficulties in multi-indicator balancing, and poor adaptive capability, struggles to cope with large-scale complex scheduling scenarios; the latter, plagued by the explosion of the decision variable space, faces bottlenecks such as premature convergence, local optimality, difficulty in convergence, and a sharp decline in scheduling efficiency with the expansion of task scale. Against this backdrop, AI technology enables end-to-end decision-making modeling through data-driven approaches, breaking the dependence of traditional methods on explicit rules or fixed operators, improving computational efficiency for complex mapping relationships, and thus providing a new paradigm for solving cataloging task scheduling problems.

### 4.4. AI-Based Method

To date, AI methods have not been directly applied to the field of cataloging task resource scheduling. However, advances in related domains such as measurement and control resource scheduling offer valuable references for this direction. To improve both the solution quality and the efficiency of scheduling problems, researchers have explored multiple application paradigms of AI algorithms in resource scheduling [54,55,56,57]. An overview of these AI-based resource scheduling paradigms, including their categories, meanings, and characteristics, is provided in Table 7.

In summary, AI algorithms can learn complex mappings from large-scale data, providing effective support for guiding algorithm evolution or directly generating scheduling solutions, thereby opening new research avenues for cataloging task resource scheduling. However, challenges such as the difficulty of acquiring large-scale training data, the black-box nature of models, and security risks arising from output instability [66] remain critical issues that must be carefully considered when applying these methods in the aerospace domain.

## 5. Multi-Objective Optimization-Based Conventional Cataloging Resource Scheduling

Real-world space object surveillance scheduling problems typically involve multiple conflicting optimization objectives, such as task completion rate, resource load balancing, and response timeliness. Therefore, the essence of this problem lies in being a typical multi-objective optimization problem, whose core task is to provide decision makers with a comprehensive perspective for systematically trading off among different performance metrics. However, existing research still exhibits clear limitations in its optimization paradigms: most studies tend to focus on a single objective, either solely pursuing task completion rate or only concerning resource utilization efficiency. Even when multiple objectives are considered, they are commonly simplified into a single-objective problem via linear weighting, which fails to genuinely reconcile the inherent conflict between task execution effectiveness and resource utilization rationality, thereby limiting the overall improvement of the system’s comprehensive performance.

Against this backdrop, multi-objective optimization methods offer a feasible path toward more scientific and efficient scheduling. Within the multi-objective optimization framework, if a solution is no worse than another in all objectives and strictly better in at least one, it is said to Pareto-dominate the latter. The set of all solutions that are not dominated by any other constitutes the Pareto-optimal set, which delineates the best achievable trade-off frontier among the various objectives. Introducing multi-objective optimization algorithms shifts resource scheduling from seeking a single “optimal solution” toward analyzing and understanding the entire objective space, thereby providing more systematic and flexible decision-making support for practical applications.

### 5.1. Multi-Objective Evolutionary Algorithms

Multi-objective optimization problems in real-world applications are often NP-hard, making them difficult to solve with traditional algorithms. In contrast, evolutionary algorithms possess unique advantages for tackling such problems due to their population-based search strategy and ability to obtain multiple feasible solutions in a single optimization run. As shown in Table 8, multi-objective evolutionary algorithms (MOEA) can be categorized into three types based on their selection mechanisms: Pareto dominance-based MOEAs, decomposition-based MOEAs, and indicator-based MOEAs.

### 5.2. Applications of Multi-Objective Optimization Algorithms in the Field of Resource Scheduling

To date, relatively few studies have directly adopted the multi-objective optimization theoretical framework to address cataloging task resource scheduling; however, numerous exploratory achievements have been made in related fields such as Tracking, Telemetry, and Command (TT&C) task scheduling and air and missile defense radar resource allocation. Existing research primarily focuses on balancing multiple performance indicators under complex conditions, such as multi-radar collaboration, task priority conflicts, and energy and time constraints, thereby highlighting the potential advantages of multi-objective optimization algorithms in complex resource scheduling.

The key challenge of resource scheduling based on multi-objective optimization lies in balancing the convergence and diversity of the solution set, thereby providing decision makers with diverse candidate solutions including optimal ones. To enhance the search capability and efficiency of algorithms, some researchers have adopted algorithm fusion or operator improvement strategies, such as developing a hybrid adaptive genetic algorithm by integrating chaotic sequences and an elite retention strategy to simultaneously improve the importance, urgency, and timeliness of tasks [76]; combining crossover and mutation strategies with Multi-Objective Particle Swarm Optimization (MOPSO) to boost global search capability [77]; introducing conflict resolution and tabu search mechanisms into the Multi-Objective Evolutionary Algorithm (MOEA) framework to effectively balance task failure rate and load balancing [78]; and fusing simulated annealing with discrete particle swarm optimization to improve global search ability and robustness [74]. Additionally, integrating domain knowledge into multi-objective optimization can also effectively guide the search and enhance efficiency. Examples include compressing the search space through “circle-arc” decoupling [79] and improving NSGA-II with greedy node selection to strengthen convergence and diversity [80]. However, such methods have limited improvement in global optimization capability, and the quality of generated solutions heavily depends on the rationality of domain knowledge, making them prone to insufficient diversity maintenance and suboptimal global optimization performance.

To ensure solution set diversity, some researchers have incorporated adaptive and learning mechanisms into multi-objective optimization. Reference [75] proposes a Multi-Objective Differential Evolutiont (MODE) algorithm based on space partitioning and an adaptive selection strategy: the space partitioning strategy maintains population diversity, while the adaptive resource allocation mechanism accelerates the optimization process of promising subspaces, achieving a favorable balance between convergence and diversity. Such algorithms excel in solution quality; however, the efficiency issue arising from their high computational complexity has become a key bottleneck restricting their application in the resource scheduling field.

Overall, multi-objective optimization-based space target surveillance resource scheduling currently faces challenges such as the difficulty in balancing convergence and diversity caused by high-dimensional decision spaces, as well as low computational efficiency. In addition, the rational selection of Pareto solution sets poses higher requirements for decision makers’ professional competence. In the future, key research directions will focus on improving algorithms’ convergence capability and diversity maintenance ability in solving cataloging task resource scheduling problems through approaches such as algorithm fusion, integration of domain knowledge, and adaptive learning mechanisms, and considering the construction of an interactive decision support interface to enhance the interpretability and usability of Pareto solution sets.

## 6. Discussion and Future Works

In recent years, with the rapid development of space activities worldwide, the number of on-orbit space targets has grown exponentially, significantly escalating the risk of orbital collisions. Against this backdrop, ground-based surveillance radar networks, as the core of the space target surveillance system, are confronted with increasingly arduous, complex, and dynamic surveillance requirements. Therefore, researching intelligent resource scheduling strategies to improve task completion performance and the rationality of resource utilization in cataloging task scenarios has become critical to enhancing the overall effectiveness and robustness of the surveillance system.

To comprehensively evaluate the performance of the compared algorithms in space object cataloging scenarios, a set of simulation experiments is constructed as follows:

The scheduling horizon spans 24 h from 04:00 on 10 November 2025 to 04:00 on 11 November 2025. The surveillance targets are low Earth orbit objects, with the specific quantity determined by the scheduling scenario, covering both active spacecraft and cataloged space debris. Each space object corresponds to a surveillance task: within the scheduling horizon, if any radar in the radar network successfully tracks an object and the tracking duration meets its minimum dwell time, the task is considered successful. The minimum dwell time for each task is determined by its priority level, with the specific correspondence detailed in Table 9.

The experimental scenario includes nine ground-based radars deployed at representative sites. Among them, Radars 1 to 4 are phased-array radars, and Radars 5 to 9 are mechanical radars. All radars share a uniform minimum elevation threshold of 5°. Parameters that directly affect resource scheduling (i.e., multi-target tracking capacity and state transition time) are listed in Table 10. Other radar parameters (such as azimuth and elevation limits and detection range) are only used to generate visibility windows via STK and have no direct impact on the scheduling model. For brevity, these are not included in Table 10.

The feasibility of tracking a space object by a radar is determined by their visibility. In visibility assessment, the Earth’s curvature is adopted as the primary constraint; additional occlusion effects such as terrain and ground objects are not considered at this stage and are reserved for future work. A continuous period during which a space object is visible to a ground-based radar is recorded as a visibility time window. Within the scheduling horizon, orbital propagation of space objects is performed using the SGP4 model, and all visibility time windows between space objects and the ground-based radar network are precomputed using the Satellite Tool Kit (STK). Through data preprocessing, a set of candidate time windows for each task is obtained. An example set of candidate time windows for selected tasks is provided in Table 11.

In this paper, the cataloging task scale is divided into four levels (with space object quantities of 400, 1000, 4000, and 8000), denoted as Scenario 1 to Scenario 4. The task completion rate, total priority gain of completed tasks, and load imbalance degree are selected as algorithm evaluation metrics, with their calculation methods given in Equations (6)–(10). Among them, the task completion rate and total priority gain are benefit-type objectives (larger values indicate better task completion performance), while the load imbalance degree is a cost-type objective (smaller values reflect more rational resource utilization and a superior scheduling scheme).

(6) $$f_{1}=\frac{\sum_{i=1}^{V}x_{i}}{V}$$

(7) $$f_{2}=\sum_{i=1}^{V}x_{i}\cdot p_{i}$$

(8) $$f_{3}=\frac{\sigma }{\mu }$$

(9) $$\sigma =\sqrt{\frac{1}{N}\sum_{j=1}^{N}{\left(y_{j}-\bar{y}\right)}^{2}}$$

(10) $$\mu =\max\left\{y_{j}|j=1,2,\ldots ,N\right\}$$

Here, the symbols in the formulas are defined as follows:

$x_{i}$ Completion status of task *i*: 1 means the task is successfully scheduled, and 0 means the task fails to be scheduled;
*V* 
Total number of cataloging tasks to be scheduled;
$p_{i}$ Priority of task *i* (ranging from 1 to 10, where a higher $p_{i}$ value indicates greater task importance);
$y_{j}$ Resource utilization rate of radar *j* during the scheduling cycle;
$\bar{y}$ Average resource utilization rate of all radars in the scheduling cycle;
*N* 
Total number of radars.

Five algorithms were selected for resource scheduling across the above four scaled scenarios, including a heuristic algorithm based on random allocation rules [9], a load balancing-oriented heuristic algorithm [5], a priority-based sorting heuristic algorithm, the NSGA-II, and the Learning-Guided Nondominated Sorting Genetic Algorithm II (LGNSGAII) [12] (denoted as Algorithm 1, Algorithm 2, Algorithm 3, Algorithm 4, and Algorithm 5, respectively). The specific parameter settings of each algorithm are shown in Table 12. All algorithm parameters were empirically determined through preliminary sensitivity analysis on representative instances and fixed globally across all scenarios. The scheduling time was measured on a single thread using MATLAB 2020b’s tic/toc functions. Experiments were conducted on an Intel Core i7-11800H CPU @ 2.30 GHz with 32 GB RAM running Windows 10.

Table 13 presents one representative scheduling solution (Solution 1) obtained by Algorithm 5 under Scenario 4, providing a concrete example of how tasks are assigned to radars with specific time windows. As shown, each successfully scheduled task is allocated to a radar with a designated start and end time, and the scheduled duration satisfies the task requirement.

Table 14 presents the performance comparison of five algorithms across four task scales. As Algorithms 4 and 5 are multi-objective optimizers that produce a Pareto front, the table lists performance metrics for three representative solutions selected from each Pareto front per scenario. These solutions were chosen for their diversity in the objective space to characterize the solution set. The results indicate that all algorithms can achieve near-optimal solutions for small-scale tasks. However, as the task scale increases, Algorithm 4 (which relies solely on metaheuristic search) and Algorithm 1 (based on random allocation rules) both exhibit significant performance degradation. In particular, Algorithm 4, which lacks any domain knowledge, struggles to generate feasible scheduling schemes. In contrast, heuristic methods based on load-balancing rules (Algorithm 2) and priority rules (Algorithm 3) demonstrate notable advantages in resource utilization rationality and task completion quality, producing clearly superior schedules in terms of load balancing and overall benefit guarantee. Nevertheless, both heuristic approaches adopt arc segments as the basic processing units. When confronted with large-scale tasks, the number of arc segments grows sharply, leading to a quadratic increase in computational complexity and a substantial rise in scheduling time, even exceeding that of iterative metaheuristic algorithms.

**Figure 6:**
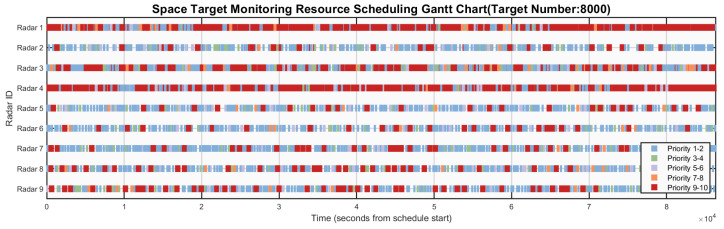
Gantt chart of algorithm 1’s scheduling scheme in scenario 4.

**Figure 7:**
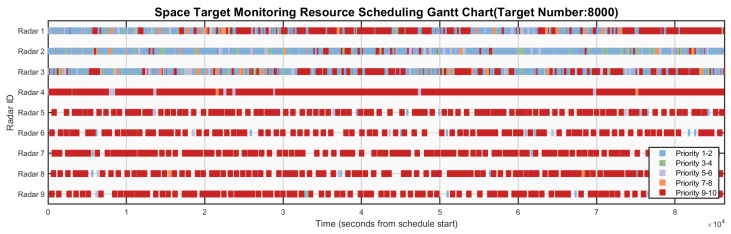
Gantt chart of algorithm 2’s scheduling scheme in scenario 4.

**Figure 8:**
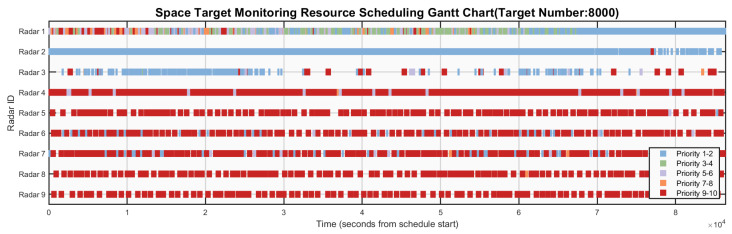
Gantt chart of algorithm 3’s scheduling scheme in scenario 4.

**Figure 9:**
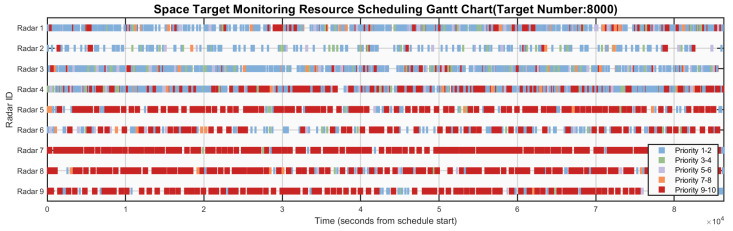
Gantt chart of algorithm 4’s scheduling scheme in scenario 4.

**Figure 10:**
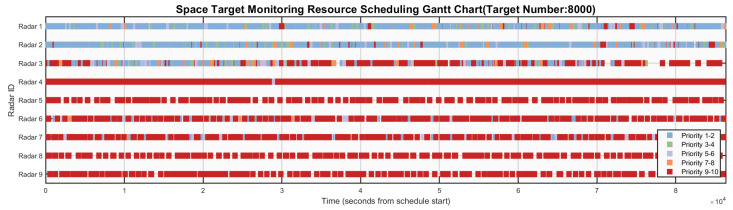
Gantt chart of algorithm 5’s scheduling scheme in scenario 4.

Figure 6, Figure 7, Figure 8, Figure 9 and Figure 10 respectively present the scheduling results of Algorithms 1–Algorithms 5 in Scenario 4. From the comparative analysis of the scheduling schemes, it can be observed that Algorithm 4 exhibits the worst overall performance. Its task allocation and execution sequence are almost completely random, resulting in a large amount of idle resources on Radars 1–4, which possess strong multi-target surveillance capabilities, while these radars are assigned numerous low-value tasks. This leads to severe misalignment in resource allocation and highlights a prominent issue of resource waste. Although Algorithm 1 also adopts a random allocation mechanism, its greedy strategy for selecting task execution time slots improves resource utilization efficiency to some extent. Therefore, despite the randomness in task-resource matching, the final scheduling scheme of Algorithm 1 is still superior to that of Algorithm 4. Among the remaining three algorithms with relatively better performance, Algorithm 3 tends to prioritize high-priority tasks but lacks sufficient consideration of performance differences across radar types. This is evidenced by the excessive load on Radars 6 and 7, while Radar 3 (which possesses stronger multi-target surveillance capability) remains underutilized, indicating a clear deficiency in the rationality of task allocation. This not only creates an imbalance characterized by coexistence of resource idleness and over-occupation but also poses potential risks to the long-term stable operation of Radars 6 and 7. In contrast, Algorithm 2 fully demonstrates its adaptability to radar heterogeneity, achieving the most reasonable resource allocation and the best load balancing performance, with its scheduling scheme exhibiting the best overall effectiveness. Algorithm 5 also achieves satisfactory scheduling results, demonstrating well-rounded comprehensive performance.

The above analysis demonstrates that the incorporation of heuristic rules significantly benefits the solution of large-scale optimization problems such as cataloging task resource scheduling, and the integration of heuristic rules with evolutionary algorithms can effectively improve the overall quality of scheduling schemes. Therefore, exploring more sophisticated heuristic rules that comprehensively consider task priorities and radar heterogeneity, and effectively integrating them with evolutionary algorithms, represents an important direction for future improvement.

**Figure 11:**
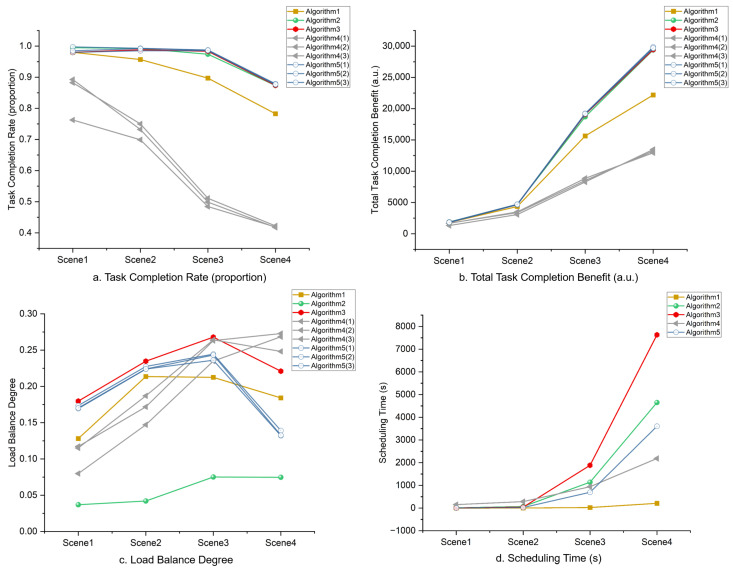
Performance metrics of scheduling schemes across four tasks in Scenario 4.

Figure 11 illustrates the performance trends of the five algorithms across varying task scales. In terms of scheduling quality, Algorithms 2, 3, and 5 all perform well, with Algorithm 5 showing a slight advantage in task completion rate and total gain. Regarding the key metric of load balance, Algorithm 2 consistently outperforms the others, although its balance index exhibits a gradual decline as the task scale increases—reflecting the inherent limitations of greedy strategies under tight constraints. In contrast, Algorithm 1 displays non-monotonic fluctuations from 400 to 8000 tasks, highlighting the complex interplay between randomness and statistical regularity.

Computational efficiency varies markedly among the algorithms. Algorithm 1 is the fastest but yields unsatisfactory scheduling quality. Algorithms 2 and 3, which process massive numbers of arc segments, suffer from quadratic growth in computational complexity, leading to a sharp rise in runtime as the scale expands and thus limiting their scalability. By contrast, Algorithm 5 employs a layered architecture that decouples task sequencing from resource allocation: the upper layer uses a metaheuristic to optimize the task order, while the lower layer applies efficient heuristic rules for resource matching. This design fundamentally reduces the search space, enabling Algorithm 5 to maintain high-quality schedules while effectively avoiding an explosion in computational cost.

In summary, Algorithm 1 is efficient but ineffective; Algorithms 2 and 3 produce high-quality solutions but are difficult to scale; and Algorithm 5, through its layered cooperative mechanism, achieves a balance among scheduling quality, load balance, and computational efficiency in all scenarios with more than 400 tasks, demonstrating strong potential for handling larger-scale problems. These results validate the effectiveness of the layered design philosophy that “decouples task sequencing from resource allocation and applies metaheuristic search and domain-rule guidance separately”.

**Figure 12:**
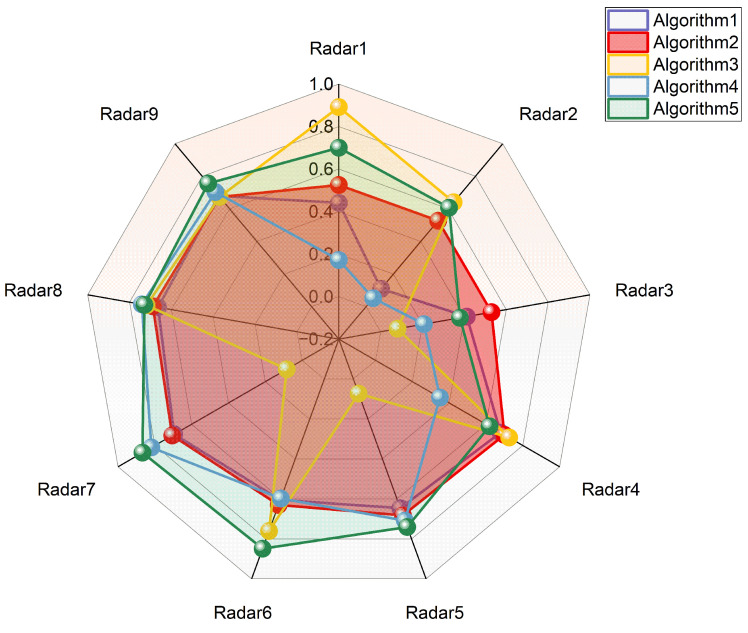
Resource utilization rate of each radar for five algorithms in Scenario 4.

Figure 12 illustrates the resource utilization rate of each radar in the five scheduling schemes obtained under Scenario 4. It can be observed that the scheme generated by Algorithm 2 maintains a stable utilization rate of around 60% across all radars, representing the most balanced and rational task allocation. Algorithm 5, which adopts hierarchical optimization, follows closely, achieving fairly reasonable task allocation on eight of the radars (excluding Radar 3). In contrast, the schedules produced by Algorithms 3 and 4 exhibit highly uneven resource distribution: some radars reach nearly 80% utilization, while others remain below 20%, resulting in both resource idleness and unfulfilled tasks due to expired time windows. In summary, incorporating load balancing guidance into the search process can effectively improve the rationality of task allocation; meanwhile, the hierarchical optimization based Algorithm 5 also demonstrates outstanding performance in terms of load balancing.

To further analyze the performance differences between the two metaheuristic algorithms on this problem, five classic multi-objective optimization metrics (Inverted Generational Distance (IGD) [81], Hypervolume (HV) [67], Spacing (SP) [82], Epsilon Indicator (E) [83], and Maximum Spread (MS) [84]) are adopted to evaluate the quality of their solution sets. (In the implementation, the reference set for IGD, E, and MS is constructed by merging all solutions obtained by the five algorithms under each experimental scenario and extracting their nondominated front. The reference point for HV is set to the nadir point in the normalized objective space. All objective values are normalized prior to metric calculation: task completion rate and total revenue are transformed into cost-type objectives within [0, 1] via Equations (11) and (12), while the load imbalance degree, which inherently lies in [0, 1], is directly used as a cost-type objective without further conversion. Additionally, internally dominated solutions are removed from the solution sets of Algorithms 4 and 5 before computing the metrics to ensure fairness and accuracy.) Among them, IGD and HV are used to comprehensively assess the convergence and diversity of the solution sets: a smaller IGD value and a larger HV value indicate better overall performance. SP measures the uniformity of the distribution of solutions in the objective space, where a smaller SP value corresponds to a more uniform and better distributed solution set. The E indicator quantifies the minimum distance by which a solution set needs to be shifted to dominate the reference set; a smaller E value indicates better convergence. MS (Maximum Spread) reflects the extent of spread of the solution set in the objective space, with a larger MS value denoting wider coverage and better diversity. Table 15 presents the evaluation results of the two algorithms’ solution sets under the four scenarios using these five metrics.

(11) $$f_{1}^{\prime }=1-f_{1}$$

(12) $$f_{2}^{\prime }=1-\frac{f_{2}}{\sum_{i=1}^{V}p_{i}}$$

This section presents a comparative analysis between NSGA-II (Algorithm 4) and LGNSGAII (Algorithm 5) across four task scenarios with varying scales. The results indicate that, in the small-scale Scenario 1, NSGA-II benefits from a broader search range and superior solution diversity, outperforming LGNSGAII in key metrics such as IGD, HV, ϵ, and MS. It is capable of generating more scheduling schemes with favorable load balancing performance. In contrast, LGNSGAII suffers from insufficient exploration of the optimal solution space and is prone to local optima, resulting in relatively inferior overall performance. Scenario 2 serves as a transitional phase: while NSGA-II maintains advantages in some metrics, its HV value is overtaken by LGNSGAII, signaling the onset of performance divergence as task complexity increases. As the task scale further expands to Scenarios 3 and 4, NSGA-II, lacking embedded domain knowledge, performs blind search within the vast solution space, leading to a sharp deterioration in IGD, HV, and other metrics, and struggles to deliver feasible scheduling solutions. In contrast, LGNSGAII, empowered by domain knowledge, achieves efficient exploration and successfully outperforms NSGA-II, demonstrating its greater suitability for complex scheduling optimization scenarios.

**Figure 13:**
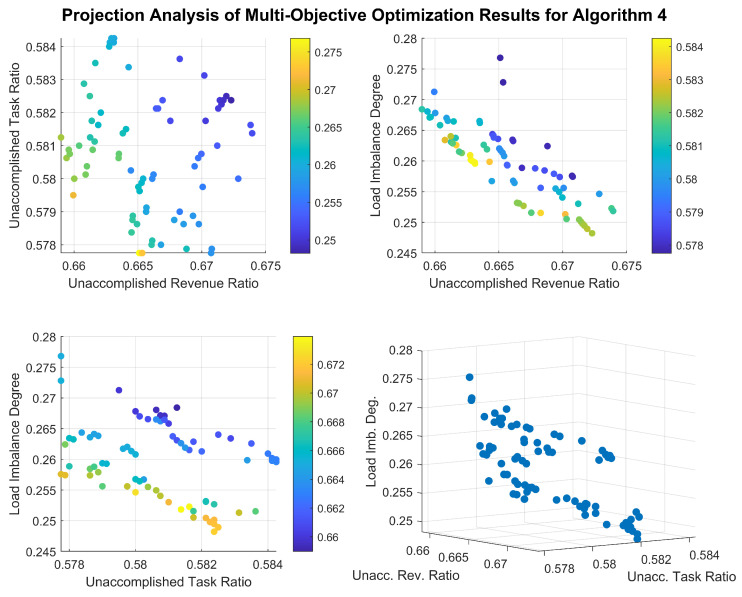
Distribution of the Solution set obtained by Algorithm 4 in Scenario 4.

**Figure 14:**
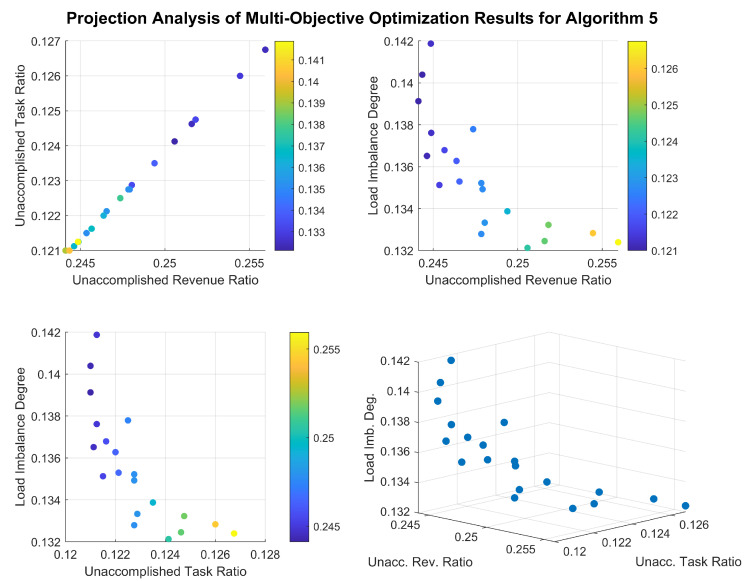
Distribution of the solution set obtained by Algorithm 5 in Scenario 4.

Figure 13 and Figure 14 illustrate the distribution of the solution sets obtained by the two algorithms in Scenario 4. To eliminate the influence of different scales among the optimization objectives, the task completion rate and total gain of completed tasks have been transformed into the task incompletion rate and the proportion of uncompleted task gain to the total gain, thereby normalizing all three indicators. It can be observed that the final solution set of Algorithm 4 contains a large number of inferior and dominated solutions, and none of the three indicators have reached a converged state. In contrast, although Algorithm 5 can obtain solutions of good quality, its solution set distribution is relatively concentrated. Furthermore, a clear linear positive correlation exists between task completion rate and total gain in its optimization results, indicating that its ability to maintain solution diversity needs improvement.

**Figure 15:**
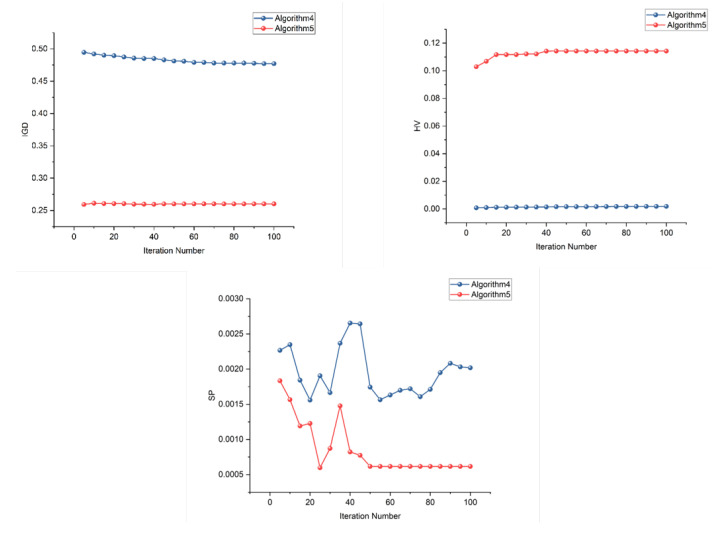
Iterative trend of the metrics for the two metaheuristic algorithms in Scenario 4. (Since the two algorithms are configured with different numbers of iterations, the metric values of Algorithm 5 after the 50th iteration are represented by its results at the 50th iteration).

Figure 15 illustrates the iterative trends of the three metrics for the two algorithms in Scenario 4. It can be observed that, in this scenario, the hierarchical optimization based Algorithm 5 demonstrates superior performance from the very beginning of the evolution and maintains a significant advantage throughout the iterative process. Its performance is particularly outstanding on the IGD and HV metrics, which comprehensively evaluate both convergence and diversity. This highlights the strong capability of its domain knowledge embedded hierarchical optimization framework in improving solution set quality and accelerating convergence. However, it is also noteworthy that both metaheuristic algorithms show relatively limited overall improvement in the IGD and HV metrics, indicating that their convergence capability and global optimization performance on this problem still require considerable enhancement.

To further validate the effectiveness of the hierarchical optimization framework and to eliminate the influence of fundamental algorithmic differences, we conducted an additional comparative experiment involving three classical multi-objective evolutionary algorithms (MOEAs): Multi-Objective Differential Evolution (MODE), Multi-Objective Particle Swarm Optimization (MOPSO), and NSGA-II. These algorithms were evaluated on Scenarios 2–4 using the same three optimization objectives and scheduling time as performance metrics. For each algorithm, we selected the three scheduling schemes that exhibited the best overall performance in terms of diversity and convergence from the obtained solution sets, and present their objective values in Table 16.

The results, summarized in Table 16, reveal that the performance of MODE, MOPSO, and NSGA-II is remarkably similar across all three scenarios, and all of them are significantly outperformed by Algorithms 2 and 5 reported earlier. This finding reinforces a key insight of our study: for large-scale optimization problems characterized by complex constraints and a vast solution space, pure evolutionary algorithms alone struggle to produce high-quality schedules. Instead, effective integration of problem-specific heuristic rules with evolutionary search (such as exemplified by Algorithms 5) is essential to achieve superior performance. The close performance of the three generic MOEAs further confirms that the observed gap is not due to the choice of a particular evolutionary paradigm, but rather to the lack of domain knowledge embedded in the solution process.

These observations not only reinforce the above insight, but also reveal a common bottleneck shared by all tested algorithms: as task scale increases, their scheduling performance declines significantly and computational time rises sharply. With the continued expansion of space activities globally, the number of space objects will grow rapidly, and task scales will keep expanding, placing higher demands on the convergence capability, global optimization efficiency, and solving speed of algorithms. Therefore, how to effectively cope with the continuously expanding solution space and ensure that algorithms can still balance scheduling effectiveness and operational efficiency in large-scale task scenarios has become a key challenge in this field.

In summary, resource scheduling for cataloging tasks is evolving from static, rule-based allocation towards adaptive, multi-objective collaborative, and intelligent scheduling. Based on the comparative experiments and analysis above, we suggest that future research should focus on the following three critical directions to address the dual challenges of continuously growing task scales and increasing demands for real-time responsiveness:

1. Development of highly scalable and efficient scheduling algorithms for massive space object scenarios. Current algorithms generally experience a significant performance drop when the number of tasks exceeds 4000. Future work should explore novel algorithm frameworks based on problem decomposition, hierarchical collaboration (e.g., “global screening followed by local refinement”), or data-driven surrogate models. The goal is to manage the explosion of the decision space and achieve sub-linear growth in algorithm runtime relative to task scale.
2. Development of strongly adaptive and robust scheduling mechanisms for open and complex environments. Existing research is predominantly validated in static and deterministic environments. Future studies need to construct scheduling models that incorporate uncertainties such as the sudden appearance of new targets, unexpected radar failures, and dynamic drastic changes in task priorities. Furthermore, it is essential to develop robust algorithms equipped with online learning and fast rescheduling capabilities to ensure system stability under disturbances.
3. Construction of an interpretable and interactive decision support system facilitating multi-dimensional trade-offs. Multi-objective optimization generates a large number of Pareto-optimal solutions, which places a significant decision-making burden on commanders. Future research should investigate technologies such as Pareto front visualization, solution difference tracing, and preference injection. The aim is to build a human-machine collaborative decision-making closed loop that can efficiently translate the solution set output by algorithms into executable decisions, thereby enhancing the practical utility of the system.

## 7. Summary and Conclusions

This study systematically reviews the resource scheduling technologies for ground-based radar networks oriented to cataloging tasks. The core insight is that this problem is essentially a large-scale, sparse solution space, multi-objective NP-hard optimization problem constrained by multiple stringent constraints, and its technical evolution is shifting from single-rule scheduling to an intelligent collaborative scheduling paradigm integrating domain knowledge. The main contributions of this study are as follows:

1. Systematic Synthesis and Modeling Analysis: This study organizes and synthesizes the optimization objectives, complex constraints, and two core decision-making models (priority assignment and conflict resolution) involved in this field, thereby contributing to a more structured understanding of the problem’s foundational elements.
2. Analysis of Algorithmic Evolution and Performance: This work systematically analyzes the evolutionary trajectory and application paradigms of algorithms in both single-objective and multi-objective optimization contexts. Building on this, by establishing a unified benchmark framework for multi-task scenarios, it conducts the first direct comparison of the scheduling performance and efficiency of mainstream algorithms under a consistent experimental setup. This clarifies their performance degradation patterns as task scale increases, providing practical guidance for algorithm selection in real-world applications.
3. Pathways for Integrated Innovation: Experimental results demonstrate the effectiveness of the domain knowledge-embedded hierarchical optimization framework in balancing scheduling quality and efficiency. Furthermore, this study proposes concrete future research directions, such as integrating AI and enhancing decision-support systems, thereby outlining potential pathways for advancing large-scale scheduling capabilities.

The limitations and future work of this study are as follows: First, the review scope is focused on conventional cataloging tasks, with insufficient coverage of dynamic rescheduling scenarios such as emergency response and high-maneuver target tracking. This is mainly due to the lack of unified evaluation benchmarks for dynamic scenarios in existing studies, which limits the systematic comparison of related algorithms. Second, although the experimental verification is carried out in multi-scale scenarios, there is still a gap with the complexity and uncertainty of real massive targets (e.g., tens of thousands of targets), especially in terms of the dynamic change characteristics of target trajectories and the randomness of equipment faults.

Future research should seek breakthroughs in three directions: scalable algorithms for ultra-large-scale scenarios, robust adaptive scheduling in open dynamic environments, and human-machine collaborative intelligent decision making. In particular, exploring discrete adaptations of differential evolution—a powerful evolutionary paradigm that has shown promise in other optimization domains—for this combinatorial NP-hard problem constitutes a valuable future direction. These efforts aim to advance the transformation of this technology from theoretical methods to engineering applications, thereby effectively ensuring the efficient and stable operation of space situational awareness systems.

## Figures and Tables

**Table 1:** Cataloging-oriented resource scheduling constraints.

Category	Constraint Name	Description
Spatiotemporal and Geometric Constraints	Visibility Constraint	Geometric visibility and minimum elevation angle, as well as other performance constraints, must be satisfied between space targets and surveillance resources.
Physical and Capability Constraints of Equipment	State Transition Time Constraint	Sufficient time must be reserved between two adjacent tasks assigned to the same resource to realize the switching of equipment states.
	Multi-Target Surveillance Capability Constraint	Determined by the radar’s beamforming capability and the hardware count of transmit/receive channels, the number of space targets assigned to a radar shall not exceed its multi-target surveillance capability.
	Energy Resource Constraint	To ensure equipment safety and performance, the radar’s instantaneous and average transmit power must be strictly limited within its design-specified power envelope.
	Processor Resource Constraint	Determined by the computational capacity and capability of the radar’s backend signal processor and data processor, the total number of channels occupied by various tracking tasks shall not exceed the radar’s physical and processing limits.
Task Requirement Constraints	Task Time Window Constraint	Tracking shall start after the earliest allowed start time of the task and end before the latest allowed end time.
	Task Duration Constraint	The tracking duration shall not be less than the task duration specified in the requirements.
	Tracking Frequency Constraint	The actual tracking frequency of each task shall not be less than the tracking frequency specified in the requirements.

**Table 2:** Optimization objectives for cataloging task resource scheduling in space target surveillance.

Optimization Objective Type	Optimization Objective	Description
Task Satisfaction	Task Completion Rate	The ratio of the number of completed tasks to the total number of tasks.
	Number of Completed Tasks	The total number of successfully completed tasks.
	Critical Task Completion Rate	The ratio of the number of completed critical tasks to the total number of critical tasks.
	Total Priority Score	The sum of priorities of all successfully scheduled tasks within one scheduling cycle.
Resource Utilization	Load Balancing Degree	The average level of utilization rates among all surveillance resources within one scheduling cycle.
	Resource Utilization Rate	The ratio of the resource’s service time to its available service time within one scheduling cycle.
	Ground Station Priority	Priority weights are set based on the inherent attributes of ground stations, and scheduling strategies are adopted to ensure that high-priority ground stations preferentially undertake suitable tasks.

**Table 3:** Factors influencing priority.

Category	Metric	Relationship with Priority (Inherent Logic and Direction of Influence)
Target Attributes	Orbital Altitude	Lower altitude leads to more significant perturbative effects (e.g., atmospheric drag) and faster orbital decay, necessitating more frequent tracking to maintain catalog accuracy. (Positive)
	Strategic Affiliation	For own core space assets and non-own associated space entities, continuous monitoring is conducted to maintain the safe and stable operation of own space assets, while supporting a sound understanding of space situational awareness and avoiding unnecessary risks to own assets. (Positive)
	Radar Cross Section (RCS)	A smaller RCS increases difficulty in detection and tracking. Higher priority is assigned to maintain situational awareness of such low-observable targets and ensure mission success. (Negative)
	Object Function	Owned critical assets or non-cooperative/potentially threatening objects require continuous monitoring to ensure own security and understand others’ intentions. (Positive)
	Time Since Launch	A more recently launched object has greater uncertainty regarding its function and precise orbital behavior, posing a higher potential risk. (Negative)
	Remaining Design Lifetime	Objects nearing their design end-of-life have diminished orbital maintenance capability and a higher probability of malfunction or breakup. (Negative)
	Physical Size	Larger objects pose a greater debris-generation hazard upon collision and are often high-value or high-threat targets themselves. (Positive)
Task Attributes	Task Type	Emergency tasks (e.g., collision warning, re-entry prediction) directly concern space safety and typically have higher priority than routine cataloging tasks.
	Remaining Time to Deadline	Task urgency increases dynamically as its available time window shrinks, serving as a core input for dynamic priority models. (Negative)
System Status	Time Since Last Observation	To prevent loss of track and maintain catalog integrity, the priority of an object should gradually increase the longer it remains unobserved. (Positive)

A positive correlation indicates that a larger value of the metric corresponds to a higher priority; a negative correlation indicates the opposite.

**Table 4:** Classification of conflict resolution methods.

Name	Meaning	Characteristics
Selection-Elimination Method	When facing a conflict, decide which window to keep based on certain rules (e.g., priority, urgency), and delete the others.	Simple to operate, but requires reasonable rule setting. Additionally, it often suffers from low efficiency, poor global optimality, and load imbalance.
Conflicting Window Pruning Method	Based on window priority, prune the conflicting portions of lower-priority windows, retaining only the conflict-free parts that exceed the minimum tracking window length.	Can utilize resources more fully and improve task completion rate. However, its effectiveness highly depends on prior knowledge and pruning rules; improper settings may lead to the erroneous removal of critical information.
Conflict Backtracking Class	Re-select tasks by backtracking through already-scheduled windows to resolve increased conflict rates caused by unreasonable time window assignments.	Possesses recovery capability, is more likely to achieve global optimality, is suitable for dynamic environments, and can be combined with other conflict handling methods. However, it has high computational cost and complex state management and may get stuck in loops or lose partial progress.

**Table 5:** Evolution of heuristic algorithms for cataloging task scheduling.

Category	Meaning	Usage Examples	Characteristics
Static Priority Rules	Guide scheduling using fixed, pre-defined criteria without real-time adjustments.	Sort by target importance (descending) [6]; random allocation based on resource availability [9].	Simple and effective for ensuring critical tasks in low-conflict scenarios; lacks global optimality as problem scale expands.
Multi-Rule Combination	Integrate multiple complementary rules to balance conflicting objectives (e.g., efficiency vs. stability).	Incorporate load balancing for radar network stability [5]; fuse rules to improve resource utilization and task completion rate [46].	Mitigates local optimality issues; however, achieving a reasonable trade-off among conflicting rules remains a key bottleneck.
Dynamic Priority Mechanisms	Generate priorities online by synthesizing real-time task and environment information.	Synthesize threat level, dwell time, and deadlines [8,47,48].	Transforms scheduling into an adaptive process; suitable for dynamic environments and time-sensitive tasks; aligns with real-world command logic.

**Table 6:** Improvement strategies for metaheuristic algorithms in cataloging task scheduling.

Category	Meaning	Usage Examples	Characteristics
Algorithm Fusion	Balance search depth and breadth by combining complementary strengths of different algorithms to avoid premature convergence.	GA + Simulated Annealing (avoids premature convergence) [36]; GA + Tabu Search (strengthens fine-grained search) [49].	Effectively balances convergence speed and solution quality; simple to implement but still constrained by the limitations of base algorithms.
Feature-Based Improvement	Leverage task characteristics (e.g., clustering) or adaptive mechanisms to reduce problem scale or enhance local search.	Dynamic Task Clustering + SA [50]; Adaptive VNS with Metropolis Criterion and Tabu List [51].	Highly targeted and improves convergence; however, computational efficiency decreases linearly with task scale, limiting scalability for large catalogs.
Hierarchical Optimization Framework	Decouple into “task sequence optimization” and “resource allocation” layers; metaheuristics search sequences, domain knowledge decodes feasible solutions.	Metaheuristics optimize priority sequences, decoded by domain-aware time window selection algorithms [12,52,53].	Reduces decision space dimensionality, ensures feasible solutions, and boosts convergence speed; requires careful design of inter-layer coupling mechanisms.

**Table 7:** Classification of AI-based resource scheduling approaches.

Category	Meaning	Usage Examples	Characteristics
AI-Metaheuristic Hybridization	Leverage the data-driven nature of AI to guide or accelerate the evolutionary search process of metaheuristic algorithms (e.g., genetic algorithms).	1. Adjust GA operators via Gated Recurrent Unit (GRU)-based RL network [58]; 2. Assist GA with DRL for feasible solution construction [59]; 3. Select schedulers adaptively via deep Q-learning [60]; 4. Apply K-means clustering + cluster-level GA operations [61]; 5. Use neural networks as surrogate models for fitness evaluation [62,63].	Combines AI’s learning capability with metaheuristics’ global search strengths, improving solution quality and computational efficiency. However, performance is bounded by base metaheuristics’ weaknesses (e.g., local optima traps).
Hierarchical Optimization Framework	Decompose the scheduling problem into hierarchical layers, assigning AI for high-level matching and traditional heuristics for low-level timing determination.	DRL for task-to-antenna matching + heuristic rules for task start/end time determination [64].	Clear division of labor; leverages mature heuristics to ensure baseline feasibility and stability. Complex design required for inter-layer coupling and coordination.
End-to-End Scheduling Generators	Directly generate complete scheduling solutions using deep learning models, often with preprocessing for decision space reduction.	1. Graph clustering + DDPG for satellite observation scheduling [14]; 2. DeepRM_Plus (RL + imitation learning) for cloud resource scheduling [65].	Superior performance in large-scale scenarios; aligns with real-world dynamic decision-making. Heavily relies on offline training, incurs high computational costs, and has limited adaptability to emergency scenarios.

**Table 8:** Classification of MOEAs.

Category	Representative Algorithms	Characteristics	Application Scenarios
Pareto dominance-based MOEAs	Strength Pareto Evolutionary Algorithm (SPEA) [67], SPEA2 [68], Nondominated Sorting Genetic Algorithm II (NSGA-II) [69].	Most widely used and robust; efficiently generates well-distributed Pareto optimal sets via fast nondominated sorting and crowding distance.	Routine cataloging scheduling requiring intuitive trade-offs among key metrics such as task completion rate, total priority gain, and load balancing.
Decomposition-based MOEAs	Multi-Objective Evolutionary Algorithm based on Decomposition (MOEA/D) and its variants [70,71,72].	High computational efficiency and strong scalability with respect to the number of objectives; however, solution quality heavily depends on the decomposition strategy and weight vector settings.	Complex scheduling scenarios with more than three objectives, where decomposition shows significant potential.
Indicator-based MOEAs	Indicator-Based Evolutionary Algorithm (IBEA) [73], Hypervolume Estimation Algorithm (HypE) [74], Reference Vector Guided Evolutionary Algorithm (RVEA) [75].	Relieve reliance on Pareto dominance and effectively address the loss of selection pressure in high-dimensional objective spaces; however, they incur high computational cost and require careful indicator design.	High-dimensional multi-objective scheduling models involving multiple conflicting objectives.

**Table 9:** Correspondence between task requirements and priorities.

Task Requirement Level	Proportion	Corresponding Priority	Track Time (s)
Low	60%	1–5	180
Medium	30%	6–8	300
High	10%	9–10	600

**Table 10:** Radar types and their key performance parameters.

Radar ID	Radar Type	Multi-Target Tracking Capability	State Transition Time (s)
1–4	Phased array radar	10	0.01
5–9	Mechanical radar	1	10

**Table 11:** Example of candidate time windows for cataloging tasks(ellipsis indicates omitted rows for brevity).

Task ID	Radar ID	Start Time (s)	End Time (s)	Duration (s)
1	1	889.1	1269.9	380.8
1	1	6950.2	7163.7	213.5
1	1	31,933.4	32,152.6	219.2
1	1	37,828.9	38,212.3	384.4
1	1	43,848.4	44,186.1	337.2
…	…	…	…	…

**Table 12:** Algorithm parameter settings.

Algorithm	Number of Iterations	Population Size	Crossover Rate	Mutation Rate
Algorithm 4	100	100	0.7	0.05
Algorithm 5	50	20	0.7	0.05

**Table 13:** Illustration of Scheduling Solution 1 obtained by Algorithm 5 in Scenario 4(ellipsis indicates omitted rows for brevity).

Task ID	Radar ID	Start Time (s)	End Time (s)	Duration (s)	Priority
1	1	78,919	79,099	180	3
2	1	17,762	17,942	180	1
…	…	…	…	…	…
14	2	47,631	47,811	180	4
16	7	77,395	77,995	600	10
18	4	41,787	42,387	600	10
…	…	…	…	…	…

**Table 14:** Algorithm performance comparison across four task scenarios.

Task Scenario	Algorithm	Task Completion Rate	Total Priority Gain of Completed Tasks	Load Imbalance Degree	t/s
Scenario 1	Algorithm 1	98.00%	1806	0.1281	0.463
	Algorithm 2	99.5%	1866	0.0369	9.845
	Algorithm 3	98.00%	1806	0.1797	4.068
	Algorithm 4	89.25%	1661	0.1151	153.967
		76.25%	1313	0.0799	
		88.25%	1688	0.1172	
	Algorithm 5	99.75%	1876	0.1736	6.398
		98%	1806	0.1694	
		98.5%	1826	0.1703	
Scenario 2	Algorithm 1	95.70%	4353	0.2136	1.511
	Algorithm 2	99.25%	4707	0.0420	66.567
	Algorithm 3	98.80%	4662	0.2347	40.380
	Algorithm 4	73.20%	3487	0.1870	284.272
		69.90%	3063	0.1471	
		75%	3374	0.1718	
	Algorithm 5	99.3%	4712	0.2275	23.163
		98.5%	4632	0.2239	
		99.2%	4702	0.2240	
Scenario 3	Algorithm 1	89.70%	15,629	0.2125	23.203
	Algorithm 2	97.40%	18,704	0.0751	1140.094
	Algorithm 3	98.35%	19,085	0.2679	1883.116
	Algorithm 4	49.88%	8855	0.2642	939.724
		48.45%	8288	0.2349	
		51.10%	8518	0.2632	
	Algorithm 5	98.75%	19,242	0.2445	695.232
		98.53%	19,153	0.2361	
		98.68%	19,213	0.2432	
Scenario 4	Algorithm 1	78.26%	22,194	0.1841	208.556
	Algorithm 2	87.33%	29,394	0.0747	4651.150
	Algorithm 3	87.40%	29,453	0.2210	7630.952
	Algorithm 4	41.76%	12,946	0.2482	2185.012
		41.88%	13,472	0.2684	
		42.23%	13,221	0.2728	
	Algorithm 5	87.90%	29,861	0.1391	3603.801
		87.59%	29,606	0.1321	
		87.73%	29,714	0.1328	

**Table 15:** Multi-objective algorithm performance indicators.

Scenario	Algorithm	IGD	HV	SP	E	MS
Scenario 1	NSGA-II	${\mathrm{9.8723 \times 10}}^{-3}$	${\mathrm{1.9395 \times 10}}^{-1}$	${\mathrm{5.7764 \times 10}}^{-3}$	${\mathrm{5.6472 \times 10}}^{-2}$	${\mathrm{9.0733 \times 10}}^{-1}$
	LGNSGAII	${\mathrm{2.1051 \times 10}}^{-1}$	${\mathrm{1.0823 \times 10}}^{-1}$	${\mathrm{4.3611 \times 10}}^{-3}$	${\mathrm{2.6140 \times 10}}^{-1}$	${\mathrm{1.4813 \times 10}}^{-1}$
Scenario 2	NSGA-II	${\mathrm{1.7795 \times 10}}^{-2}$	${\mathrm{2.7334 \times 10}}^{-2}$	${\mathrm{3.4706 \times 10}}^{-3}$	${\mathrm{9.0549 \times 10}}^{-2}$	${\mathrm{3.7881 \times 10}}^{-1}$
	LGNSGAII	${\mathrm{3.7493 \times 10}}^{-1}$	${\mathrm{9.9066 \times 10}}^{-2}$	${\mathrm{8.7487 \times 10}}^{-3}$	${\mathrm{3.3166 \times 10}}^{-1}$	${\mathrm{5.7674 \times 10}}^{-2}$
Scenario 3	NSGA-II	${\mathrm{4.9551 \times 10}}^{-1}$	${\mathrm{1.8618 \times 10}}^{-3}$	${\mathrm{2.0187 \times 10}}^{-3}$	${\mathrm{6.0549 \times 10}}^{-1}$	${\mathrm{5.0090 \times 10}}^{-2}$
	LGNSGAII	${\mathrm{2.5388 \times 10}}^{-1}$	${\mathrm{1.1427 \times 10}}^{-1}$	${\mathrm{1.1131 \times 10}}^{-3}$	${\mathrm{5.5035 \times 10}}^{-1}$	${\mathrm{2.3772 \times 10}}^{-2}$
Scenario 4	NSGA-II	${\mathrm{6.3773 \times 10}}^{-1}$	0	${\mathrm{8.0980 \times 10}}^{-4}$	${\mathrm{8.3040 \times 10}}^{-1}$	${\mathrm{4.6279 \times 10}}^{-1}$
	LGNSGAII	${\mathrm{5.3312 \times 10}}^{-2}$	${\mathrm{8.5913 \times 10}}^{-3}$	${\mathrm{1.1509 \times 10}}^{-3}$	${\mathrm{7.2858 \times 10}}^{-1}$	${\mathrm{1.2292 \times 10}}^{-1}$

**Table 16:** Performance comparison of MODE, MOPSO, and NSGA-II on Scenarios 2–4.

Scenario	Algorithm	Task Completion Rate	Total Priority Gain of Completed Tasks	Load Imbalance Degree	Time (s)
Scenario 2	MODE	67.40%	3078	0.1475	104.035
		74.10%	3303	0.1748	
		73.60%	3362	0.1770	
	MOPSO	69.40%	3067	0.1736	63.651
		68.60%	3061	0.1738	
		67.80%	2947	0.1615	
	NSGA-II	73.20%	3487	0.1870	284.272
		69.90%	3063	0.1471	
		75.00%	3374	0.1718	
Scenario 3	MODE	45.33%	7746	0.2336	478.716
		52.60%	8710	0.2606	
		51.83%	8826	0.2615	
	MOPSO	48.38%	8107	0.2452	438.301
		46.03%	7817	0.2344	
		46.85%	7956	0.2433	
	NSGA-II	49.88%	8855	0.2642	939.724
		48.45%	8288	0.2349	
		51.10%	8518	0.2632	
Scenario 4	MODE	39.09%	12,325	0.2535	1405.287
		45.93%	13,463	0.2717	
		45.29%	13,549	0.2713	
	MOPSO	39.69%	12,143	0.2583	1915.823
		41.04%	12,743	0.2602	
		40.85%	12,684	0.2642	
	NSGA-II	41.76%	12,946	0.2482	2185.012
		41.88%	13,472	0.2684	
		42.23%	13,221	0.2728	

## Data Availability

No new data were created or analyzed in this study.

## Abbreviations

The following abbreviations are used in this manuscript:

LEO	Low Earth Orbit
GEO	Geosynchronous Earth Orbit
AI	Artificial Intelligence
RCS	Radar Cross Section
MADM	Multi-Attribute Decision Making
AHP	Analytic Hierarchy Process
MIP	Mixed-Integer Programming
GRU	Gated Recurrent Unit
DRL	Deep Reinforcement Learning
DeepRM_Plus	Deep Resource Management Plus
DDPG	Deep Deterministic Policy Gradient
SPEA	Strength Pareto Evolutionary Algorithm
NSGA-II	Nondominated Sorting Genetic Algorithm II
IBEA	Indicator-Based Evolutionary Algorithm
HypE	Hypervolume Estimation Algorithm
RVEA	Reference Vector Guided Evolutionary Algorithm
TT&C	Tracking, Telemetry, and Command
MOPSO	Multi-Objective Particle Swarm Optimization
MOEA/D	Multi-Objective Evolutionary Algorithm based on Decomposition
MOEA	Multi-Objective Evolutionary Algorithm
MODE	Multi-Objective Differential Evolutiont
LGNSGAII	Learning-Guided Nondominated Sorting Genetic Algorithm II
IGD	Inverted Generational Distance
HV	Hypervolume
SP	Spacing
